# Prevalence and Determinants of the Pool Sign in Lung Cancer Patients with Brain Metastasis

**DOI:** 10.2174/0115734056401497250904223250

**Published:** 2025-10-02

**Authors:** Ying Long, Zhao-ping Chen, Lin-hui Wang, Xue-qing Liao, Ming Guo, Zhong-qing Huang

**Affiliations:** 1 Department of Medical Image Center, Yuebei People’s Hospital, Shantou University Medical College, Shantou, China; 2 Department of Pathology, Yuebei people’s Hospital, Shantou University Medical College, Shantou, China; 3 Department of Neurosurgery, Yuebei people’s Hospital, Shantou University Medical College, Shantou, China

**Keywords:** Metastasis, Brain, Pool sign, Lung cancer, Magnetic resonance imaging

## Abstract

**Purpose:**

The pool sign, an emerging MRI biomarker for differentiating brain metastases (BM) from primary neoplasms, is primarily documented in case reports. Systematic data on its prevalence and determinants in BM among patients with lung cancer are lacking. This study aims to evaluate the occurrence of the pool sign and identify factors associated with its presence.

**Materials and Methods:**

Between January 2017 and August 2024, data from 6,004 lung cancer patients were retrospectively extracted from the electronic health records system. The clinical and demographic characteristics, along with BM MRI features, were compared between the pool sign and non-pool sign groups using univariate and multivariate analyses.

**Results:**

A total of 427 patients (81 women; mean age, 62.17 years) were enrolled in the study. The pool sign was observed in 29 patients (6.8%). The inter-reader reliability for the pool sign ranged from moderate to substantial (κ=0.61–0.80), while the intra-reader reliability was moderate (κ=0.6). In the univariate analysis, a statistically significant difference was observed in the volume size of metastases between the pool sign group and the non-pool sign group (median 4.8 *vs*. 0.5, *P* < 0.0001). This finding suggests that the presence of the pool sign is more likely associated with BMs exhibiting relatively larger tumor volumes. Additionally, the prevalence of solid-cystic masses was significantly higher in the pool sign group compared to the non-pool sign group, with rates of 79.3% and 44.5%, respectively (*P* = 0.0014). However, there were no statistically significant differences in other examined variables. In the multivariate analysis, the findings demonstrated that an increase in tumor volume (OR = 1.050, 95% CI 1.025-1.076, *P* < 0.001) and the presence of a solid-cystic mass (OR = 3.666, 95% CI 1.159-11.595, *P* = 0.027) were significantly correlated with a higher probability of pool sign occurrence.

**Conclusion:**

The pool sign occurs in 6.8% of BM in patients with lung cancer and is independently associated with larger lesion volume and solid-cystic morphology. Its diagnostic utility warrants further validation.

## 1 BACKGROUND

Brain metastasis (BM) develops in patients with lung cancer at an incidence rate ranging from 23% to 36%, constituting the most prevalent intracranial metastatic tumor [1-3]. The occurrence of BM events is indicative of a poor prognosis, with a median survival time of 3 to 6 months, presenting a substantial clinical challenge [4].

The “pool sign” was first reported in 2018 by Alok A et al. [5] and has been identified as a potentially easy-to-use biomarker for differentiating metastases from primary neoplasms, as evidenced by three subsequent reports [6-8]. The sign manifests as a perilesional hyperintense rim on T2-weighted imaging (T2WI), situated between the solid tumor margin and vasogenic edema [5, 6, 8]. It demonstrates greater conspicuity and reliability on T2WI compared to T1-weighted imaging (T1WI), where signal characteristics are variable (typically isointense to hypointense relative to gray matter) [5].

Although the pool sign has been utilized in clinical diagnosis, to our knowledge, no prior study has comprehensively investigated its prevalence and associated determinants specifically in patients with lung cancer-derived BM. We therefore aimed to systematically assess the occurrence of this imaging biomarker and its influencing factors in BM originating from lung cancer within a single institutional cohort.

## 2 MATERIALS AND METHODS

### 2.1 Patients

This retrospective study received approval from the local institutional review board at Yuebei People's Hospital (ethics code: YBSKY-2024-093-001), Shantou University Medical College, China. Given the retrospective design of the study, the requirement for informed consent was waived. Between January 2017 and August 2024, a total of 6,004 patients were identified from the electronic health records system using the keyword “lung cancer”. The inclusion criteria were as follows: (1) Patients with pathologically confirmed lung cancer; (2) Completion of brain MRI; (3) Imaging or pathology-confirmed BM; and (4) Absence of other primary tumors (e.g., thyroid cancer, breast cancer, or bowel cancer). The exclusion criteria were as follows: (1) Absence or low-quality brain MRI; (2) Insufficient data, including brain MRI obtained exclusively from external hospitals and incomplete MRI scans (e.g., absence of T2WI or T1WI with contrast sequences); (3) No detectable intracranial nodules or masses; and (4) Duplicated entries due to longitudinal follow-up. Clinical and demographic characteristics, including gender, age, smoking history, and lung cancer pathology type, were collected from the electronic health record system. Fig. (**1**) illustrates patient recruitment.

### 2.2 Immunohistochemistry and Molecular Analysis

Mutation analysis of EGFR, ALK, ROS1, KRAS, NRAS, BRAF, PIK3CA, HER2, and RET was conducted on formalin-fixed, paraffin-embedded tissue samples utilizing the Amplification Refractory Mutation System Polymerase Chain Reaction (ARMS-PCR). The ARMS-PCR assay was executed using the 2× Hieff Unicon® Multiplex ARMS qPCR Kit (Yeasen Biotech Co., Ltd., China), following DNA extraction from the tissue samples. Whenever feasible, formalin-fixed, paraffin-embedded tissues obtained at the time of tissue sampling were requested for molecular testing.

### 2.3 Image Acquisition

All examinations underwent a brain MRI protocol on 1.5 Tesla or 3 Tesla scanners (Signa HDxt, Signa Discovery 750w, GE Healthcare, and Magnetom Vida or Skyra, Siemens). The clinical brain MRI protocol, designed to evaluate suspected tumors at our institution, comprised the following sequences: axial and/or coronal diffusion-weighted imaging (DWI), axial and/or coronal fluid-attenuated inversion recovery sequence, axial T2WI and T1WI sequences, contrast-enhanced coronal and sagittal (or axial) T1WI following a single intravenous dose of a gadolinium agent, and a volumetric axial T1-weighted sequence acquired 5-7 minutes post-gadolinium injection. All images were retrieved from the picture archiving and communication system and subsequently de-identified for further analysis.

### 2.4 Definition of the Pool Sign

The pool sign must exhibit the following 5 distinct MRI features: (1) T2WI: a region of higher signal intensity than the surrounding edema band, located between the tumor margin and the oedema band. Morphology may be irregular, ring-shaped, or arc-shaped. (2) T1WI: iso- to hypo-intensity relative to gray matter (generally higher than cerebrospinal fluid). (3) DWI: absence of diffusion restriction. (4) Contrast-enhanced T1WI: absence of enhancement. (5) Location: exclusively within brain parenchyma, excluding lesions originating from the cranial bone or meninges (e.g., meningiomas and meningeal anomalies). The pool sign and its mimics are illustrated in Figs. (**2** and **3**).

### 2.5 Image Analysis and Interpretation

The images were independently reviewed by two radiologists with 13 and 15 years of experience, respectively, with no access to the clinical data and molecular data. Discrepancies were resolved by consensus. All measurements were obtained at the dedicated scanner workstation, utilizing electronic calipers with appropriate magnification. Tumor volume was calculated using the abc/2 formula [9, 10]. BM were classified into three distinct types based on post-contrast T1WI enhancement patterns at the largest lesion slice: (1) solid lesion, characterized by the enhanced component constituting ≥80% of the tumor at the slice with the largest lesion cross-section; (2) cystic lesion, the cystic (non-enhanced) component comprising ≥80% of the lesion; and (3) cystic-solid lesion, representing an intermediate state between the solid and cystic conditions.

### 2.5.1 Reproducibility Assessment

A randomly selected cohort of 100 patients underwent evaluation of the pool sign by three independent readers (Y.L., Z.C., and Z.H.) to determine inter-observer reproducibility. Intra-observer reliability was assessed through repeated evaluation by the reader (Z.C.) after a minimum of 90-day interval to mitigate recall bias.

### 2.6 Statistical Analysis

All statistical analyses were conducted using GraphPad Prism 8 and SPSS 25 software. Inter-observer agreement regarding the pool sign was analyzed using Cohen’s kappa coefficient, with values ≤ 0.40 indicating fair agreement, 0.41–0.60 indicating moderate agreement, 0.61–0.80 indicating substantial agreement, and 0.81–1.00 indicating almost perfect agreement [11]. Data following a normal distribution were presented as mean ± standard deviation (x±SD), with comparisons between the pool sign group and non-pool sign groups conducted using the independent samples t-test. For data not following a normal distribution, the Mann-Whitney U test was employed. Categorical variables were expressed as frequencies or percentages (n/%) and analyzed using the chi-square or Fisher’s exact test. A multivariate logistic regression model, utilizing the enter method, was applied to determine the odds ratio (OR) and 95% confidence interval (CI) for independent factors associated with the presence of pool sign. The model's goodness-of-fit was assessed using the Hosmer-Lemeshow test. P-values <0.05 were considered statistically significant.

## 3 RESULTS

### 3.1 Patient Characteristics

A total of 427 patients were recruited in our study, among whom the pool sign was observed in 29 (6.8%) patients. The distribution of the pool sign across brain regions revealed the following prevalence: parietal lobe (27.6%, n=8), frontal lobe (24.1%, n=7), temporal lobe (17.2%, n=5), cerebellum (10.3%, n=3), and occipital lobe (20.7%, n=6). The mean age of patients was 60.6 ± 9.2 years in the pool sign group and 63.8 ± 11.8 years in the non-pool sign group, with no statistically significant difference (*P* > 0.05) (Table **1**). Tumor volume demonstrated a non-normal distribution; the pool-sign group exhibited significantly larger metastases (median 4.8 *vs*. 0.5 cm^3^, *P*< 0.0001). Further analysis found that 14 cases (48.3%) of BM with a volume greater than 10 cm^3^ were observed in the pool sign group. In contrast, 79 cases (19.9%) of BM with a volume exceeding 10 cm^3^ were identified in the non-pool sign group. These findings indicated that the pool sign was more likely associated with BM originating from lung cancer that exhibits relatively large tumor volumes (*P*=0.0003). Morphologically, the prevalence of solid-cystic masses was significantly greater in the pool sign group compared to the non-pool sign group, with rates of 79.3% and 44.5%, respectively (*P* = 0.0014). However, there were no statistically significant differences in gender, smoking history, or pathological type of primary cancer between the pool sign and non-pool sign groups (*P* > 0.05).

### 3.2 Genotyping of Primary Lung Cancers and Identification of Pool Signs

Among 427 patients, genetic testing was unavailable for 253 (59.3%), negative in 35 (8.2%), and positive in 139 (32.6%). In the pool sign group (n=29), the *EGFR* gene mutation was most common (50.0%, n=4), followed by *KRAS* (25.0%, n=2), and *ALK* and *BRAF* (12.5% each, n=1). However, there was no statistically significant difference in the presence of mutations between the pooled and non-pooled groups (*P*=0.097).

#### 3.2.1 Multivariate Analysis

A comprehensive analysis was performed to identify the factors influencing the occurrence of pool signs, utilizing multifactorial logistic regression models that incorporated variables such as age, gender, smoking history, tumor volume, and tumor manifestations. The results indicated that an increase in tumor volume (OR = 1.050, 95% CI 1.025-1.076, *P* < 0.001) and the presence of a solid-cystic mass (OR = 3.666, 95% CI 1.159-11.595, *P* = 0.027) were significantly associated with a higher likelihood of pool sign occurrence (Table **2**).

### 3.3 Reliability

The inter-reader agreement for the pool sign was moderate (κ=0.554, Z.C. *vs*. Y.L.), substantial (κ=0.775, Z.C. *vs*. Z.H.), and near-perfect (κ=0.863, Y.L. *vs*. Z.H.), respectively. The intra-reader agreement for the pool sign was moderate (Z.C., κ=0.6).

## 4 DISCUSSION

This first systematic evaluation of the pool sign of BM in patients with lung cancer revealed an incidence rate of 6.8% within our institutional cohort (n=427). Inter-reader reliability for the pool sign demonstrated moderate to substantial (κ=0.554 to 0.863), while intra-reader reliability was moderate (κ=0.6). Tumor volume and solid-cystic morphology emerged as independent predictors, with lesions exceeding 10 cm^3^ exhibiting the highest likelihood of presenting the pool sign.

**Fig. (4):**
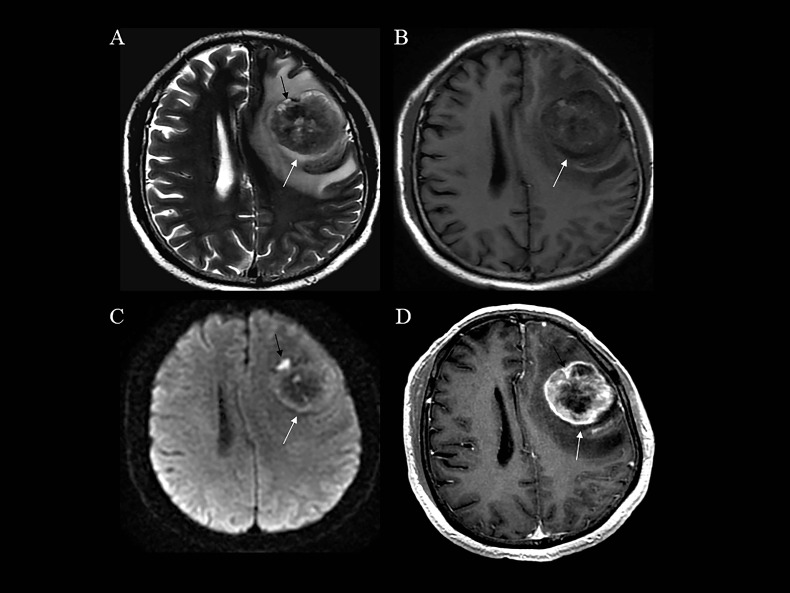
Role of DWI and contrast-enhanced T1WI in the identification of the pool sign. A perilesional T2-Weighted Imaging (T2WI) hyperintense rim (**A**, **B** white arrow) was observed between the lesion and the surrounding edema, without diffusion restriction (**C**, white arrow) and without enhancement (**D**, white arrow). This region demonstrated a true pool sign. Conversely, a similar T2WI hyperintense region was observed (**A**, black arrow), but with diffusion restriction (**C**, black arrow) and enhancement (**D**, black arrow). This corresponding area exhibited a false pool sign.

The definition of the pool sign as reported in the literature does not include contrast agent-enhanced imaging and DWI. However, these sequences are essential in the assessment of various brain pathologies, including brain tumors and tumor-like lesions, in modern neuroimaging practices [12-15]. In order to accurately identify the pool marker, and due to the rich vascular network and microenvironmental biology of metastatic tumors compared to the surrounding brain structures [16-19], these two sequences will enable us to exclude the effects of cystic and fluid necrosis of the metastatic tumor itself (Fig. **4**). Hence, we have revised the definition as follows: (i) A perilesional T2-weighted imaging hyperintense rim situated between the lesion and vasogenic edema, without diffusion restriction (DWI) and enhancement (post-contrast T1WI); (ii) The tumor must be located within the brain parenchyma, thereby excluding lesions originating from the cranial bone or meninges, such as meningiomas and meningeal anomalies (Fig. **S1**).

Contrary to classical edema-volume relationships [20], the pool sign correlated with larger lesions. The precise mechanism underlying the presence of the pool sign in BM from lung cancer remains unclear, and the specific pathological component responsible for this sign has not yet been identified. It is known that BM typically exhibit localized growth and are distinctly demarcated from the surrounding peritumoral edematous brain tissue, with the high signal on T2WI being indicative of purely vasogenic edema.

Previous studies have hypothesized that the pathological component of the pool sign may be related to the secretion of metastatic adenocarcinoma [6, 8]; however, this adenocarcinoma secretion hypothesis conflicted with our observation of the pool sign in squamous carcinoma, small cell carcinoma, and carcinoid tumor. Furthermore, the prevalence of cystic-solid tumors within the pool sign group being significantly greater than those in the non-pool sign group can be explained by the fact that cystic BM are essentially non-secretory [17, 21, 22] and characterized by a disorganized stroma with aberrant vasculature and collagen networks [23, 24].

BM represents a significant challenge in the management of lung cancer, not only due to its high incidence but also because it profoundly impacts patient survival and quality of life [25-27]. Mutations in key driver genes, including *EGFR*, *KRAS*, *TP53*, and *ALK*, exhibit high concordance (>80%) between primary lung tumor and BM [28]. Furthermore, the presence of *EGFR* mutations is linked to poor BM prognosis [29]. The incidence of the pool sign in our cohort (6.8%) is comparable to the incidence of the T2-FLAIR mismatch sign (7.2%) [30], which is recognized as a reliable imaging biomarker for IDH-mutant gliomas [31, 32]. We sought to establish the relationship between tumor mutations and the presence of the pool sign. Our results demonstrated that the *EGFR* mutation was the most prevalent mutation within the pool sign group (50.0%). However, the difference in the prevalence of this mutation between the pool sign group and the non-pool sign group did not reach statistical significance.

## 5 LIMITATIONS

This study had inherent limitations. Firstly, this study has a retrospective design, introducing potential selection bias and a sample with the lack of genetic testing in 253 cases. Secondly, multi-scanner data with variable field strengths and sequence parameters may affect the delineation of lesion and edema boundaries, BM volumetry measurements and pool sign identification. The reliability of the pool sign rather than the reproducibility of tumor volume was assessed. Thirdly, this study did not report the diagnostic value of the pool sign as a time dependent marker in the diagnosis of BM [6]. Our ongoing prospective study integrating mutation analysis and pool sign evaluation is underway to elucidate the differential diagnosis and management of solitary BM.

## CONCLUSION

The incidence of the pool sign is 6.8%, and the occurrence of this sign is independently influenced by tumor volume and manifestations. We first systematically reviewed the characterization of this imaging biomarker and developed a diagnostic framework for its clinical interpretation in BM. Prospective validation is required to determine its diagnostic utility and the prognostic value of the pool sign in the management of BM.

## Figures and Tables

**Fig. (1):**
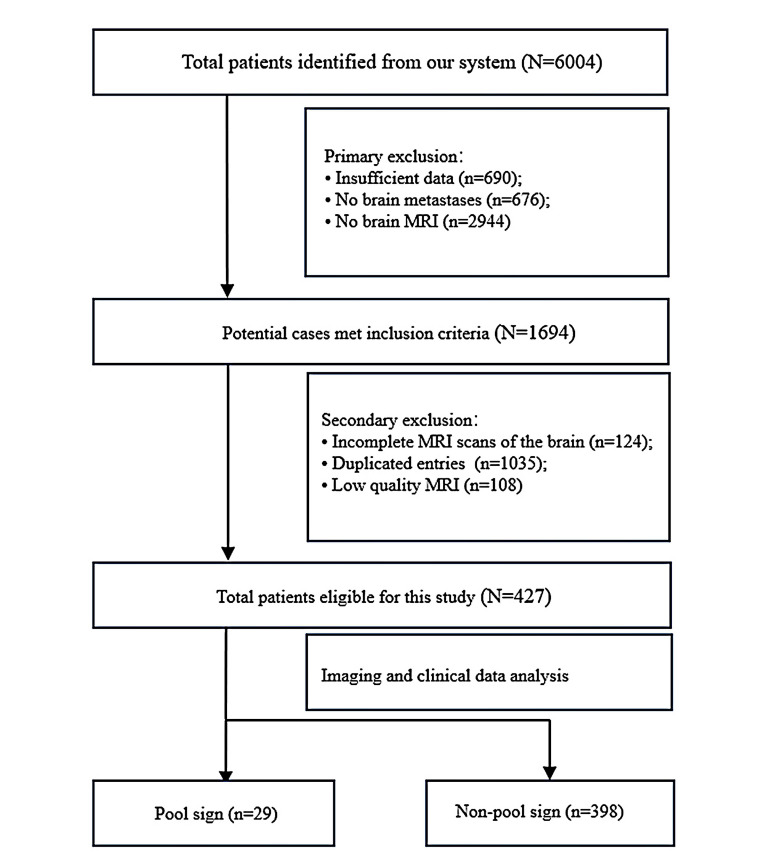
Study flowchart.

**Fig. (2):**
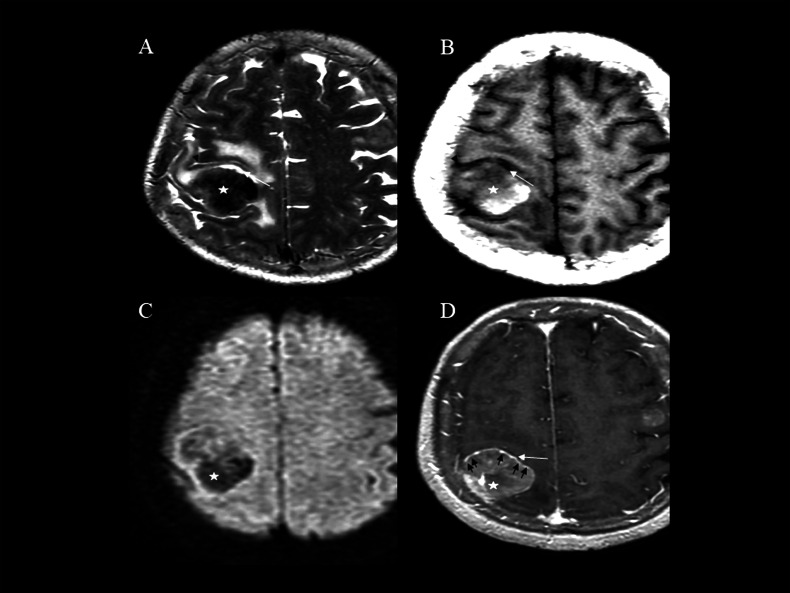
Representative case of the pool sign. (**A**): On T2WI, an arc-shaped region exhibiting higher signal intensity relative to the oedema band is observed between the tumor margin and the oedema band (white arrow). (**B**): On T1WI, the signal intensity of the corresponding area (white arrow) appears hypointense in comparison to gray matter. (**C**): On DWI, the region does not demonstrate diffusion restriction. (**D**): On contrast-enhanced T1WI, the region does not show enhancement (white arrow), although significant enhancement of the tumor's peripheral wall (black arrow) is evident. The black arrowhead indicates the cerebral sulcus between gray matter, and the star indicates the brain metastasis.

**Fig. (3):**
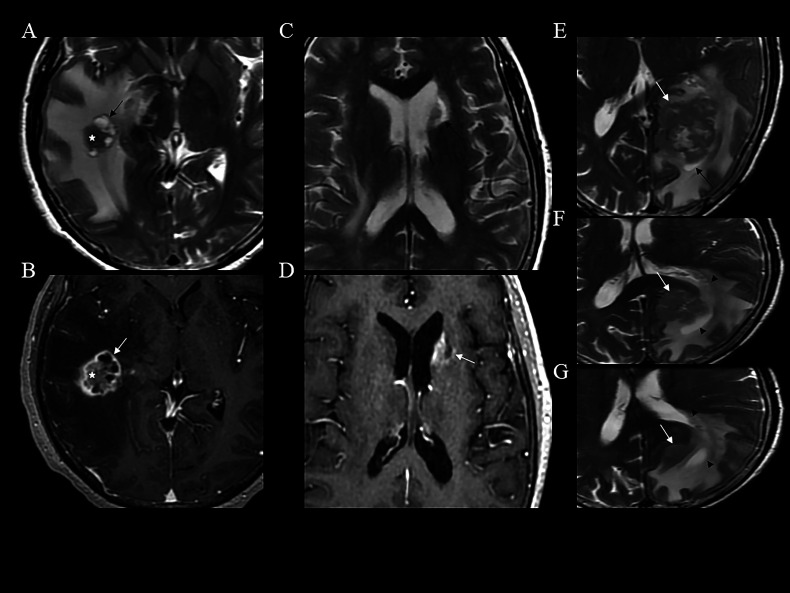
Representative images of pool sign mimics. (**A**-**B**) (Case 1): An i)rregular hyperintense area (A, black arrow) is observed at the periphery of the tumor and surrounding edema on T2WI. However, it is identified as the cystic component of the lesion on T1WI with contrast enhancement (B, white arrow). The star indicates the solid part of the tumor. (**C**-**D**) (Case 2): An a)rc-shaped region (C, black arrow) is observed relative to the parenchymal edema on T2WI. However, the corresponding region is located within the lesion edge (D, white arrow) and demonstrates strong enhancement on T1WI following the administration of a contrast agent. (**E**-**G**) (Case 3): A mass was identified *via* T2WI (white arrow). A region exhibiting a higher signal intensity relative to the surrounding peripheral edema was observed (E, black arrow). This region was subsequently confirmed to be the occipital horn of the lateral ventricle through the analysis of two consecutive images (F and G, black arrowhead). Notably, Cases 1 and 2 presented with multiple brain metastases (not shown), whereas Case 3 exhibited a solitary lesion.

**Table 1:** Patient characteristics (n, %).

**Characteristics**	**Pool sign group** **n=29**	**Non pool sign group** **n=398**	***P***
Age (years, mean ±SD)	60.55±9.15	63.78±11.83	0.1517
Sex (male/female)	19/10	227/171	0.2044
Tumour volume (Median)	4.8	0.4651	<0.0001
Tumour volume classification	-	-	0.0003
> 10 (cm^3^)	14 (48.3%)	79 (19.9%)	-
≤ 10 (cm^3^)	15 (51.7%)	319 (80.2%)	-
Smoking history	-	-	0.783
Yes	5 (17.2%)	61 (15.3%)	-
No	24 (82.8%)	337 (84.7%)	-
Solitary metastatic tumour	-	-	0.8871
Yes	8 (27.6%)	105 (26.4%)	-
No	21 (72.4%)	293 (73.6%)	-
Tumour manifestations	-	-	0.0014
Solid lesion	4 (13.8%)	150 (37.7%)	-
Solid-cystic lesion	23 (79.3%)	177 (44.5%)	-
Cystic lesion	2 (6.9%)	71 (17.8%)	-
Types of lung cancer	-	-	0.3553
Adenocarcinoma	26(89.7%)	329 (82.7%)	-
SCLC	1 (3.5%)	37 (9.3%)	-
Other types of lung cancer	2 (3.5%)	32 (8.0%)	-
Molecular typing of lung cancer mutations*	-	-	0.097
None	0 (0.0%)	35 (21.1%)	-
EGFR	4 (50.0%)	98 (59.0%)	-
ALK	1 (12.5%)	11 (6.6%)	-
Other mutations	3 (37.5%)	22 (13.3%)	-

**Abbreviation:** SCLC: small cell lung cancers; Other mutations include ALK, KRAS, BRAF, ROS1, RET, NRAS, PIK3CA, HER2; Other types of lung cancer include squamous carcinoma, carcinoid, large cell carcinoma, sarcomatoid carcinoma. * Among the 427 patients, 253 did not undergo genetic testing.

**Table 2:** Logistic regression analysis of factors influencing the occurrence of pool sign.

**Variables**	***P***	**OR (95% CI)**
Age	0.107	0.974 (0.943-1.004)
Male	0.755	0.857 (0.325-2.263)
Smoking history	0.484	1.652 (0.405-6.747)
Tumour volume size	0.000	1.050 (1.025-1.076)
Tumour manifestations	-	-
Solid masses	Reference	Reference
Solid-cystic masses	0.027	3.666 (1.159-11.595)
Cystic lesions	0.315	0.304 (0.030 - 3.103)

## Data Availability

The data and supportive information are available within the article.

## LIST OF ABBREVIATIONS

BM: Brain metastases
T2WI: T2-weighted imaging
T1WI: T1-weighted imaging
BM: Brain metastasis
MRI: Magnetic resonance imaging
DWI: Diffusion Weighted Imaging

## STANDARDS OF REPORTING

STROBE guidelines were followed.
