# Supplementary material for: Prevalence and Determinants of the Pool Sign in Lung Cancer Patients with Brain Metastasis
Source: Curr Med Imaging. 2025 Oct 2;21:e15734056401497. doi: 10.2174/0115734056401497250904223250 (PMC13223480; doi:10.2174/0115734056401497250904223250)
Supplement: Supplementary file 1 [file CMIM-21-E15734056401497_SD1.pdf]

1573-4056/25 *Send Orders for Reprints to reprints@benthamscience.net*

Current Medical Imaging

Content list available at: https://benthamscience.com/journals/cmir

Supplementary Material

Prevalence and Determinants of the Pool Sign in Lung Cancer Patients with Brain Metastasis

Ying Long[1,#], Zhao-ping Chen[1,#], Lin-hui Wang[2], Xue-qing Liao[1], Ming Guo[3,*] and Zhong-qing Huang[1,*]

[1]*Department of Medical Image Center, Yuebei People's Hospital, Shantou University Medical College, Shantou, China*
[2]*Department of Pathology, Yuebei people's Hospital, Shantou University Medical College, Shantou, China*
[3]*Department of Neurosurgery, Yuebei people's Hospital, Shantou University Medical College, Shantou, China*

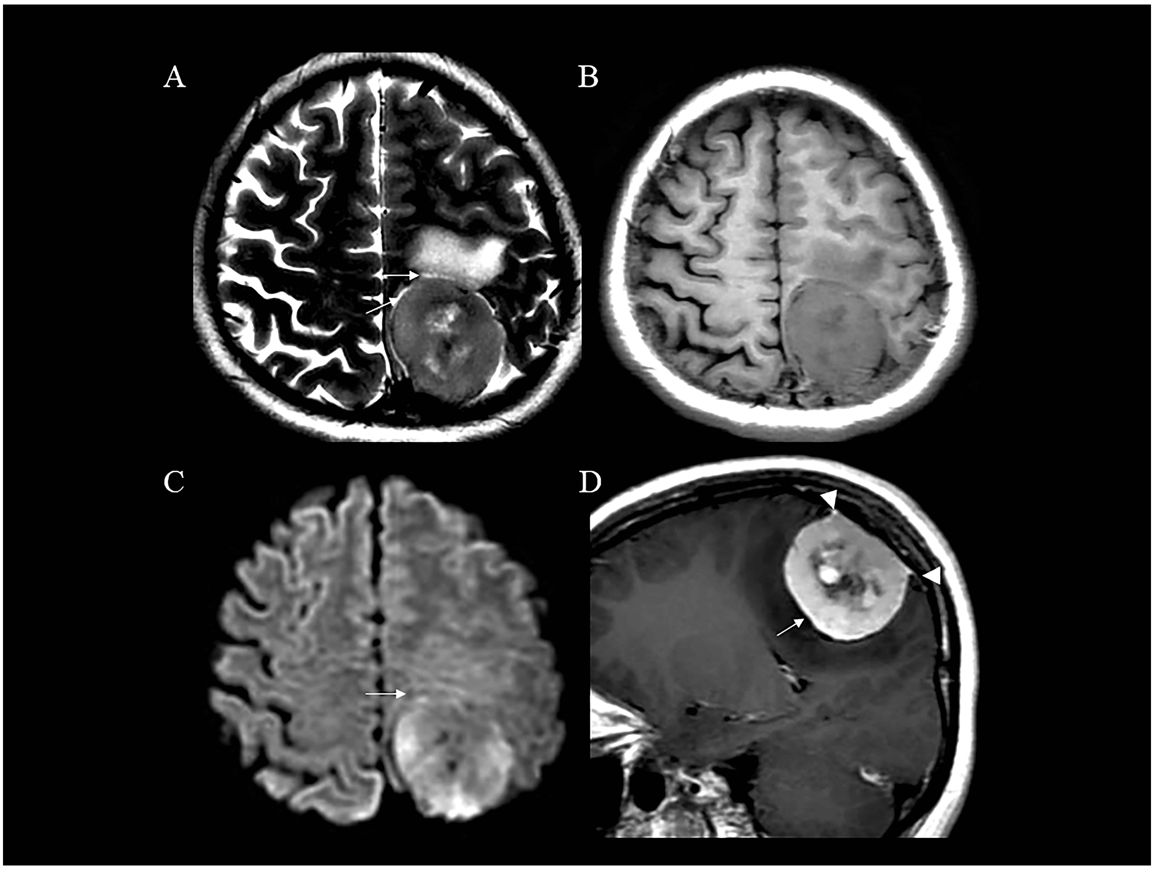

Fig. (S1). Extra-cerebral tumor mimicking pool sign. Perilesional T2WI hyperintense rim between the lesion and the surrounding edema without diffusion restriction and enhancement (white arrow) simulates pool sign. However, the presence of the meningeal tail sign suggests that the tumor is located outside the brain parenchyma (arrowhead), which was subsequently confirmed as a meningioma through pathological examination.

© 2025 The Author(s). Published by Bentham Science Publisher.

This is an open access article distributed under the terms of the Creative Commons Attribution 4.0 International Public License (CC-BY 4.0), a copy of which is available at: https://creativecommons.org/licenses/by/4.0/legalcode. This license permits unrestricted use, distribution, and reproduction in any medium, provided the original author and source are credited.

DOI: 10.2174/0115734056401497250904223250, 2025, *21*, e15734056401497
